# Investigating immune profile by CyTOF in patients with eosinophilic esophagitis after treatment with orodispersible budesonide

**DOI:** 10.1093/cei/uxae065

**Published:** 2024-07-22

**Authors:** John Plate, Sofie Albinsson Högberg, Hardis Rabe, Helen Larsson, Christine Lingblom

**Affiliations:** Department of Otorhinolaryngology Head and Neck Surgery, Region Västra Götaland, NU-Hospital Group, Trollhättan, Sweden; Department of Otorhinolaryngology, Head and Neck Surgery, Institute of Clinical Sciences, Sahlgrenska Academy, University of Gothenburg, Gothenburg, Sweden; Institute of Biomedicine, Department of Infectious Diseases, The Sahlgrenska Academy, University of Gothenburg, Gothenburg, Sweden; Institute of Biomedicine, Department of Infectious Diseases, The Sahlgrenska Academy, University of Gothenburg, Gothenburg, Sweden; RISE Research Institutes of Sweden, Bioscience and Materials, Gothenburg, Sweden; Department of Otorhinolaryngology Head and Neck Surgery, Region Västra Götaland, NU-Hospital Group, Trollhättan, Sweden; Department of Otorhinolaryngology, Head and Neck Surgery, Institute of Clinical Sciences, Sahlgrenska Academy, University of Gothenburg, Gothenburg, Sweden; Institute of Biomedicine, Department of Infectious Diseases, The Sahlgrenska Academy, University of Gothenburg, Gothenburg, Sweden; Department of Clinical Microbiology, Sahlgrenska University Hospital, Gothenburg, Sweden

**Keywords:** eosinophilic esophagitis, CyTOF, T cells, eosinophils, human, cluster analysis

## Abstract

Eosinophilic esophagitis (EoE) is a chronic Th2-mediated inflammatory disease of the esophagus driven by dietary or inhalant allergens which if left untreated, leads to fibrosis and poor esophageal function. Although the inflammation in the esophagus is dominated by eosinophils, there are also elevated levels of T and B cells. Blood samples from ten patients with EoE before and after treatment with orodispersible budesonide and 10 healthy controls were compared using cytometry by time-of-flight. An antibody panel was designed that covers the major immunological cell populations with a particular focus on eosinophils. The data was analyzed with multivariate methods and cluster analysis. Correlation analysis was done between immune markers and endoscopic, histological, and symptomatologic assessments. Our analysis revealed that patients with EoE had lower levels of effector memory T cells after treatment with orodispersible budesonide to the same level as healthy subjects. In addition, more suppressive eosinophils were present in the circulation of EoE patients before treatment and more immature eosinophils were present after treatment. Furthermore, levels of galectin-10+ eosinophils correlated with histological findings in esophageal tissue from EoE patients. In all patients, the peak eosinophils were decreased after treatment with orodispersible budesonide. Intriguingly, 90% of the patients had remission in the histological assessment and 50% improved in the endoscopic assessment. This study reports a detailed immune profile in patients with EoE before and after treatment with orodispersible budesonide and it is a step toward finding blood-based immune parameters that could be useful to monitor response to treatment.

## Teaser

(A) Minimum spanning tree of eosinophil populations present in the blood of 10 patients with EoE before and after treatment with orodispersible budesonide as well as healthy subjects (HS) determined by X-shift clustering analysis. The sizes of the circles represent the sizes of the populations. The color of the circles indicates the levels of galectin-10, FOXP3, CD16, CD274 (PD-L1), IL-5R, and IL-2R expression as shown by the heat-map scale. Numbers indicate populations that are altered after treatment [1–3 ]. Phenotypes of populations 1–3 are shown below. (B) OPLS-DA was done to see which eosinophil markers could separate EoE patients before and after treatment (*n*  = 10). (C) Loading plots with jackknife confidence intervals for the eosinophil markers are shown as boxes with ticks. The markers closely positioned to the patient categories are positively associated with the patient category in question. The generated two-component model had an explanatory power of 53% (goodness of fit R2Y = 0.53) and stability of 37% (Q2Y = 0.37).

## Introduction

Eosinophilic esophagitis (EoE) is an inflammatory disease of the esophagus i.e. considered to be driven by food or inhalant allergens [1]. It is a chronic inflammatory process in which Th2 cytokines are key mediators [2, 3], that ultimately leads to infiltration by eosinophils into the esophageal mucosa. The esophagus of healthy individuals is normally devoid of eosinophils [4]. Elevated levels of T cells, basophils, mast cells, and B cells are found in the esophagus of EoE patients [2, 3, 5, 6]. Adult EoE patients experience symptoms of esophageal dysphagia, resulting in reduced quality of life and sometimes in total food bolus obstruction. If the inflammation is left untreated it can lead to fibrosis and impaired esophageal function [7–10]. Elimination of food allergens from the diet, locally active corticosteroids, proton pump inhibitors, and dupilumab (recognizing IL-4Rα and blocking both the IL-4 and IL-13 signals) are the therapeutic options at present in Sweden [11]. Several reports demonstrate that swallowed topical corticosteroids, mainly fluticasone propionate and budesonide, are one of the most effective therapies for EoE in both children and adults [12].

Response to treatment is defined histologically by evaluating the number of peak eosinophils. A decrease below 15 eosinophils/high-power-field (HPF) is considered as remission and a decrease below five eosinophils/HPF is considered as deep remission. More recently the EoE Histology Severity Scoring (EoE-HSS) has been used [13], which is a new tool to assess more parameters of tissue inflammation in esophageal tissue samples. In addition, the Eosinophilic Esophagitis Endoscopic Reference Score (EREFS) is used which is an endoscopic scoring system for inflammatory and remodeling features of EoE including edema, rings, exudates, furrows, and stricture [14].

Suppressive eosinophils are present in the blood of healthy individuals have higher suppressive capacity and express higher levels of galectin-10 and CD16 than conventional eosinophils [7]. Suppressive eosinophils suppress all T-cell subgroups alike in a kinetic manner [15, 16] and use the intracellular protein galectin-10` in order to suppress activated T cells [17]. More recently, extracellular distribution of galectin-10 was demonstrated in the esophageal mucosa of patients with EoE, which was almost absent after treatment [18]. In the same study, CD16+ eosinophils decreased substantially after treatment [18]. Interestingly, eosinophils from patients with EoE express higher levels of the transcription factor FOXP3 and the immunoregulatory protein galectin-10 in blood compared with healthy subjects [19].

The aim of this study was to examine if it is possible to monitor response to treatment by analyzing immune markers in the blood of patients with EoE and subsequently reduce the number of repeated invasive endoscopy procedures. Thus, a blood-based analysis to follow the rate of inflammation in the esophagus would substantially increase the quality of life for the patients. Therefore, we designed an antibody panel that covers the major immune cell populations with a focus on eosinophils and analyzed the samples using mass cytometry. Cluster analysis and multivariate methods were performed to identify differences between the immune cell populations before and after treatment together with age and sex-matched controls for comparison. A second aim was to investigate the efficacy of orodipersible budesonide treatment regarding eosinophils/HPF, EoE-HSS, and EREFS, as well as symptom burden and to evaluate if any parameters correlated with immune markers.

## Materials and methods

### Study design

Twelve adult patients (≥18 years) with active EoE (≥15 eosinophils/HPF) and symptoms of esophageal dysfunction were recruited at Northern Älvsborg County Hospital (NÄL). All patients were prospectively enrolled and not selected based on response to treatment. They were offered a 12-week course of orodispersible budesonide (1 mg, 2 times per day) and their blood was analyzed before and after treatment. Previous studies have reported no systemic effect after a 12-week course of orodispersible budesonide [20]. Ten age and sex-matched controls were recruited for comparison with inclusion criteria that they had to be without esophageal symptoms. Exclusion criteria for the EoE patients were any immunomodulatory treatment, comorbidities hindering endoscopy, and failure to follow treatment protocol. Table 1 summarizes the patient demographics. The study was approved by the Regional Ethical Review Board of Gothenburg, Sweden. Written informed consent was acquired from all study participants. The study protocol conformed to the ethical guidelines of the 1975 Declaration of Helsinki.

**Table 1.** Demographics and clinical features of EoE patients

Patient ID	Age	Sex	Allergy	Type of allergy	Comorbidities	Eos/HPF (size HPF mm^2^)		Eos/mm^2^		EREFS (0–8)		EoE-Hss grade (0–1)		EoE-Hss Stage (0–1)		EEsAi Pro-score (0–100)		Watson dysphagia score (0–45)	
						Before	After	Before	After	Before	After	Before	After	Before	After	Before	After	Before	After
HE01	33	M	Yes^c^	Inhalant/food	None	37 (0.19)	0 (0.19)	195	0	6	0	0.71	0.1	0.84	0.07	50	34	36	17.5
HE02	46	F	Yes^b^	Inhalant	None	15 (0.21)	6 (0.19)	65	32	1	1	0.23	0.17	0.23	0.30	52	36	20	12
HE03	44	M	Yes^b^	Inhalant	None	32 (0.19)	22 (0.19)	168	116	3	2	0.52	0.42	0.68	0.62	58	40	21	15.5
HE04	45	M	No^b^		None	16 (0.19)	0	84	0	0	0	0.34	0.05	0.36	0.07	28	34	15	15
HE05	30	M	No^b^		None	25 (0.19)	2 (0.19)	132	11	4	4	0.52	0.1	0.51	0.19	0	0	4.5	4.5
HE06^a^	22	M	Yes^b^	Inhalant/food	Asthma	45 (0.21)	–	214	–	8	–	0.74	–	0.79	–	39	–	15	–
HE07	44	M	Yes^b^	Inhalant/food	None	24 (0.19)	0	126	0	4	4	0.42	0.21	0.65	0.23	39	27	4.5	4.5
HE08^a^	42	M	Yes^b^^,^^c^	Inhalant	None	15 (NA)	–	–	–	2	–	0.13	–	0.22	–	–	–	17.5	–
HE09	38	M	No		Sleep apnea	37 (0.21)	9 (0.19)	176	47	7	1	0.79	0.11	0.8	0.16	50	0	24.5	0
HE10	32	M	Yes^c^	Inhalant	None	52 (0.19)	0	274	0	2	1	0.32	0.14	0.46	0.25	44	35	21	14.5
HE11	67	F	No^c^		None	23 (0.19)	0	121	0	1	1	0.32	0.00	0.46	0.04	46	47	17.5	22.5
HE12	42	M	Yes^b^	Inhalant/food	Asthma	17 (0.19)	6 (0.21)	89	29	4	0	0.48	0.03	0.57	0.13	36	12	4.5	7.5

^a^Excluded from the study.

^b^RAST test.

^c^Skin prick test.

### Patient outcomes

We followed the COREOS recommended guidelines [21] analyzing the peak eosinophil count, the Eosinophilic Esophagitis Histology Scoring System (EoE-HSS), where 0 is low histological signs of inflammation and 1 is high [13], and the Eosinophilic Esophagitis Endoscopic Reference Score (EREFS) [14] with some alterations to the original score with only 8 points as recommended by the COREOS group, where 0 is low endoscopic signs of inflammation and 8 is high [21]. In addition, we utilized two questionnaires specific to EoE for evaluation of patient-reported outcome measures in dysphagia and quality of life. These two questionnaires were recently validated in Swedish i.e. the Eosinophilic Esophagitis Activity Index (EEsAI) where 0 is no burden of symptoms and 100 is a high burden of symptoms [22] and the Watson Dysphagia Scale (WDS), where 0 is no burden of symptoms and 45 is a high burden of symptoms [23]. Table 1 summarizes demographics and clinical features, and Table 2 summarizes the scoring of the EoE patients.

**Table 2.** Characteristics of EoE patients

*N* = 10	Before treatment	After treatment	*P*-value
Eosinophils/HPF (0.19–0.21 mm^2^)	28 (15–52)	5 (0–22)	0.0010
EoE-Hss grade	0.47 (0.23–0.79)	0.13 (0.00–0.42)	0.0020
EoE-Hss stage	0.56 (0.22–0.84)	0.21 (0.07–0.62)	0.0059
EREFS	3.2 (0–6)	1.4 (0–4)	0.063
Watson dysphagia score	16.9 (4.5–36)	11.4 (0–22.5)	0.078
EesAI	40.3 (0–58)	26.5 (0–47)	0.020

Numbers are mean value with min/max.

### Mass cytometry

Heparinized venous blood samples were prepared for CyTOF analysis. Erythrocytes were lyzed from heparinized venous blood samples by ammonium chloride lysis (15 min, at room temperature (RT)), and the remaining leukocytes were washed with Maxpar PBS (Fluidigm, South San Fransisco, CA, USA). Heparinized venous blood is used in order to reduce nonspecific binding between cationic proteins in eosinophils and the metal isotopes [24]. Cell suspensions were incubated with Cell ID Cisplatin (5 µm, Fluidigm, 5 min, RT), washed, and incubated with Fc receptor block Human TruStain FcX (BioLegend, San Diego, CA) and an antibody cocktail of surface markers (Supplementary Table S1) for 30 min, RT. The cells were washed, fixed in 1.6% formaldehyde solution (10 min RT), and permeabilized using Foxp3/Transcription Factor Staining buffer (eBioscience, San Diego, CA) for 1 h, RT. The cells were washed and incubated with antibodies for intracellular markers for 1.5 h, RT (Supplementary Table S1). After washing, the cells were incubated with 62.5 nm intercalation solution (Cell-ID Intercalator-Ir [125 µm], Maxpar Fix and Perm Buffer, Fluidigm) for 45 min, RT. Next, the samples were resuspended in Maxpar PBS and stored overnight at 4°C. Prior to sample analysis, the cells were resuspended in MilliQ H2O to 1 × 106 cells/ml and 0.1X EQ Four Element Calibration Beads (Fluidigm) were added. Analyses were performed using a Helios CyTOF instrument with CyTOF Software v7.0. (Fluidigm) and samples were gated using FlowJo 10.8.0 software (Tree Star Inc., Ashland, OR) (Supplementary Fig. S1). Data are presented as percentages of cells expressing the different markers.

### Multi-dimensional data analysis and statistics

CyTOF data were analyzed using X-shift clustering analysis [25]. All cells were analyzed and mapped based on 44 channels, CD3+ T cells based on 34 channels and eosinophils based on 27 channels. Pre-gated samples with the populations of interest were uploaded to the VorteX software (version 29/06/17) for X-shift clustering analysis. Multivariate analyses of pattern recognition “orthogonal projections to latent structures by means of partial least squares discriminant analysis” (OPLS-DA) were performed using the SIMCA-P (version 15.0.2) statistical package (MKS Data Analytics Solutions, Malmö, Sweden). The quality of the models was evaluated by their explanatory power (R2Y) and robustness (Q2Y). Univariate analyses regarding the expression of molecular markers and cell sub-groups were performed using GraphPad PRISM 9.2.0 software (GraphPad, San Diego, CA). Wilcoxon paired and Mann–Whitney non-paired tests were used for the comparison of the two groups, and the correlation between data sets was analyzed with Spearman’s rank correlation. *P*-values < 0.05 were considered statistically significant.

## Results

### Orodispersible budesonide is a successful treatment for patients with active EoE

Out of the 12 recruited patients, 10 patients were included in the study and 2 patients were excluded due to noncompliance with treatment. Based on histological findings, orodispersible budesonide was proven to be a successful treatment. All patients responded to the treatment although one patient did not reach remission <15 eosinophils/HPF. All patients were prospectively enrolled and not selected based on response to treatment. Eosinophils/HPF were significantly decreased from 28 eosinophils/HPF (min /max = 15/52) before treatment to 5 eosinophils/HPF (min/max = 0–22) after treatment. Three patients had remission (<15 eosinophils/HPF), six patients had deep remission (<5 eosinophils/HPF), whereas one was lowered from 32 to 22 eosinophils/HPF. In addition, the EoE-HSS stage and grade were significantly decreased after treatment. EoE-HSS stage decreased from 0.54 (min–max = 0.22–0.84) before treatment to 0.18 (min–max = 0.04–0.64) after treatment and EoE-HSS grade decreased from 0.45 (min/max = 0.13–0.79) before treatment to 0.11 (min–max = 0–0.42) after treatment. Endoscopic scoring EREFS were improved from 3.2 (min–max = 0–6) before treatment to 1.4 (min–max = 0–4) after treatment. EEsAI was improved in 7 patients with median values improving from 40.3 (min–max = 0–58) before treatment to 26.5 (min–max = 0–47) after treatment. WDS was improved in five patients whereas five patients had worsened or unchanged in their self-evaluation assessment, the median score improved from 16.9 (min–max = 4.5–36) before treatment to 11.4 (min–max = 0–22.5) after treatment. In conclusion, all patient’s peak eosinophils were decreased after treatment with orodispersible budesonide. Moreover, 90% had remission in the histological assessment, 50% improved in the endoscopic assessment, and according to the symptom scores 50% improved whereas 20% got worse. Table 2 summarizes the scoring of the EoE patients.

### Alternate levels of immune cell phenotype before and after treatment with orodispersible budesonide

**Figure 1:**
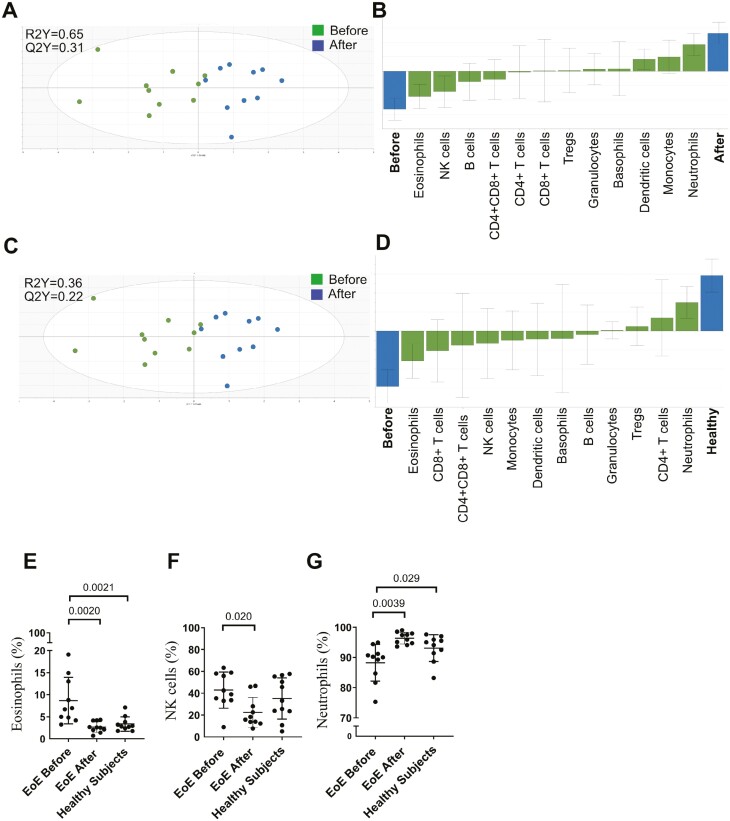
**(A)** OPLS-DA was done to see which immune cell subsets could separate EoE patients before and after treatment (*n* = 10). **(B)** Loading plots with jackknife confidence intervals for the immune cell subsets are shown as boxes with ticks. The markers closely positioned with the patient categories are positively associated to the patient category in question. The generated two-component model had an explanatory power of 65% (goodness of fit R2Y = 0·65) and stability of 31% (Q2Y = 0·31). **(C)** OPLS-DA was done to see which immune cell subsets could separate EoE patients before treatment and healthy controls (*n* = 10). **(D)** Loading plots with jackknife confidence intervals for the immune cell subsets are shown as boxes with ticks. The markers closely positioned with the patient categories are positively associated to the patient category in question. The generated two-component model had an explanatory power of 36% (goodness of fit R2Y = 0.36) and stability of 22% (Q2Y = 0.22). Univariate analysis of **(E)** eosinophils, **(F)** NK cells and **(G)** neutrophils. Data are presented as mean ± SD.

We started by making an OPLS-DA to see if we could separate EoE patients before and after treatment based on the different cell subgroups. Indeed, a model capable of separating the two groups was generated (Fig. 1A) with a robustness of 31% (Q2Y = 0.31) and with an explanatory power of 65% (R2Y = 0.65). The most discriminatory parameters are shown in Fig. 1B. On the left are the parameters associated with patients before treatment and to the right are the parameters associated with patients after treatment. Patients before treatment had higher levels of eosinophils, NK cells, and B cells and patients after treatment had higher levels of neutrophils, monocytes, and dendritic cells (Fig. 1B). Next, we constructed a model to see if we could separate EoE patients before treatment and healthy controls based on the different cell subgroups. Indeed, a model capable of separating the two groups was generated (Fig. 1C) with a robustness of 22% (Q2Y = 0.22) and with an explanatory power of 36% (R2Y = 0.36). The most discriminatory parameters are shown in Fig. 1D. On the left are the parameters associated with patients before treatment and to the right are the parameters associated with healthy controls. Patients before treatment had higher levels of eosinophils, CD8+ T cells and CD8+ CD4+ T cells and healthy controls had higher levels of neutrophils, CD4+ T cells, and Tregs (Fig. 1D). Univariate analysis confirmed that eosinophils were lower in patients after treatment to the same level as healthy subjects, and neutrophils were higher in patients after treatment to the same level as healthy subjects (Fig. 1C–E). NK cells were lowered after treatment but there was no significant difference between EoE before treatment and healthy controls. No significant difference concerning B cells and B-cell subgroups was found.

### Alternate levels of memory T cells and regulatory T cells before and after treatment with orodispersible budesonide

**Figure 2:**
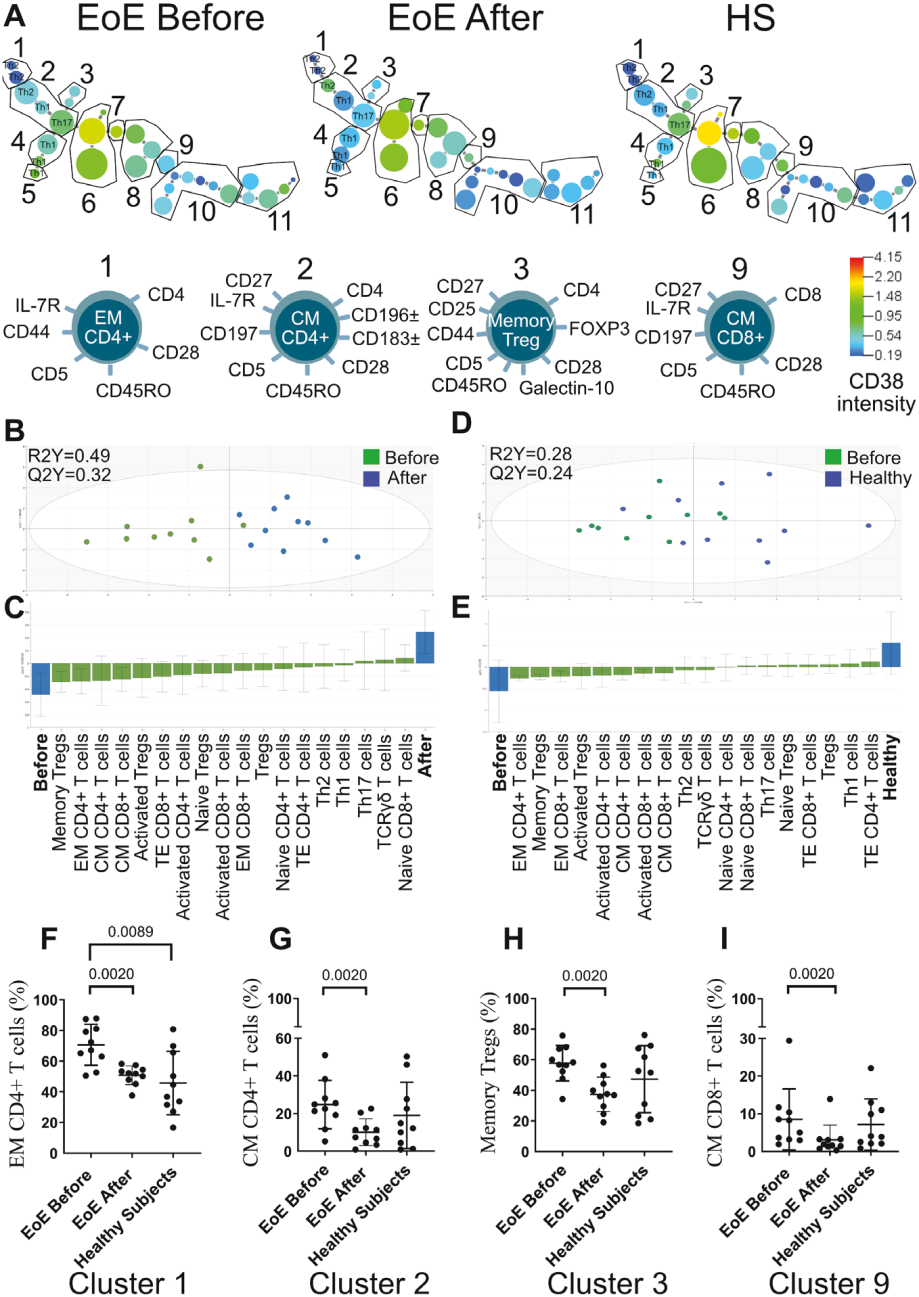
**(A)** minimum spanning tree of CD3+ T cell populations present in the blood of ten patients with EoE before and after treatment with orodispersible budesonide as well as healthy subjects (HS) determined by X-shift clustering analysis. The sizes of the circles represent the sizes of the populations. The color of the circles indicates the levels of CD38. Numbers indicate the different populations; cluster 1 = effector memory CD4+ T cells, cluster 2 = central memory CD4+ T cells, cluster 3 = memory Tregs, cluster 4 = effector memory CD4+ T cells, cluster 5 = terminal effector CD4+ T cells, cluster 6 = naive CD4+ T cells, cluster 7 = CD4+CD8+ T cells, cluster 8 = naive CD8+ T cells, cluster 9 = central memory CD8+ T cells, cluster 10 = effector memory CD8+ T cells and cluster 11 = terminal effector CD4+ T cells. Phenotypes of populations 1, 2, and 3 are shown below. **(B)** OPLS-DA was done to see which T cell subsets could separate EoE patients before and after treatment (*n* = 10). **(C)** Loading plots with jackknife confidence intervals for the T-cell subsets are shown as boxes with ticks. The markers closely positioned with the patient categories are positively associated to the patient category in question. The generated two-component model had an explanatory power of 49% (goodness of fit R2Y = 0·49) and stability of 32% (Q2Y = 0.32). **(D)** OPLS-DA was done to see, which T-cell subsets could separate EoE patients before treatment and healthy controls (*n* = 10). **(E)** Loading plots with jackknife confidence intervals for the T-cell subsets are shown as boxes with ticks. The markers closely positioned to the patient categories are positively associated to the patient category in question. The generated two-component model had an explanatory power of 49% (goodness of fit R2Y = 0.49) and stability of 32% (Q2Y = 0.32). Univariate analysis of **(F)** effector memory (EM) CD4+ T cells, **(G)** central memory (CM) CD4+ T cells, **(H)** memory Tregs, and **(I)** central memory (CM) CD8+ T cells. Data are presented as mean ± SD.

To identify how the T-cell subsets differed between EoE patients before and after treatment and healthy individuals, we continued by constructing a cluster analysis of CD3+ T cells by using the X-shift algorithm. When comparing clusters between patients with EoE before and after treatment and healthy subjects we found differences in size and intensity of the clusters for effector memory CD4+ T cells (cluster 1), central memory CD4+ T cells (cluster 2), memory T regs (cluster 3), and central memory CD8+ T cells (cluster 9) (Fig. 2A). Phenotypes of the different T-cell subgroups are shown below. The sizes of the populations can be seen in Table 3. Next, we constructed an OPLS-DA to see if we could separate the patients before and after treatment based on different T-cell subgroups. A model capable of separating the two groups was generated (Fig. 2B) with a robustness of 32% (Q2Y = 0.32) and with an explanatory power of 49% (R2Y = 0.49). The most discriminatory parameters are shown in Fig. 2C. On the right are the parameters associated with patients after treatment and to the left are the parameters associated with patients before treatment. Patients before treatment had higher levels of memory Tregs, effector memory CD4+ T cells, central memory CD4+ T cells and central memory CD8+ T cells and patients after treatment had higher levels of naïve CD8+ T cells and TCRγδ T cells (Fig. 2C). We also constructed an OPLS-DA to see if we could separate the patients before treatment and healthy controls based on different T-cell subgroups. A model separating the two groups was generated (Fig. 2D) with a robustness of 24% (Q2Y = 0.24) and with an explanatory power of 28% (R2Y = 0.28). The most discriminatory parameters are shown in Fig. 2E. On the right are the parameters associated with patients after treatment and to the left are the parameters associated with healthy controls. Patients before treatment had higher levels of effector memory CD4+ T cells, memory Tregs, and effector memory CD8+ T cells and healthy controls had higher levels of terminal effector CD4+ T cells, Th1 cells, and Tregs. Next, univariate analysis confirmed that effector memory CD4+ T cells decreased after treatment to the level of healthy controls (Fig. 2D) and central memory CD4+ T cells, memory Tregs, and central memory CD8+ T cells significantly decreased after treatment but there were no different before treatment and healthy controls (Fig. 2E–G). We did not see any significant difference concerning naïve CD8+ T cells and TCRγδ T cells before and after treatment with orodispersible budesonide.

**Table 3.** Percentage of populations of interest from the cluster analysis on CD3+ T cells

Cluster	Before treatment	After treatment	Healthy controls
1 (EM CD4+ T cells)	3.8	2.1	2.7
Th2 cells upper cluster	1.1	1.1	1.4
Th2 cells lower cluster	2.7	1	1.3
2 (CM CD4+ T cells)	20.2	16.2	19.3
Th1 cells	1.4	5.8	2.7
Th2 cells	9.5	1.9	5.4
Th17 cells	9.3	8.5	11.2
3 (memory Tregs)	1.9	1.05	2.3
Upper cluster	1	0.45	1.4
Lower cluster	0.9	0.6	0.9
9 (CM CD8+ T cells)	4.5	0.63	1.6

### Cluster analysis reveals differences in the eosinophil molecular pattern after treatment with orodispersible budesonide

**Figure 3:**
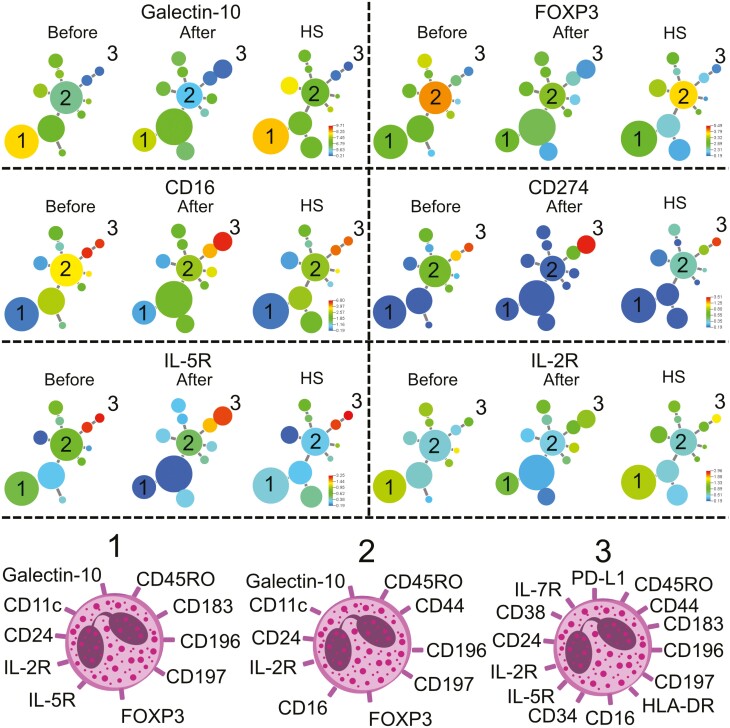
**(A)** minimum spanning tree of eosinophil populations present in the blood of ten patients with EoE before and after treatment with orodispersible budesonide as well as healthy subjects (HS) determined by X-shift clustering analysis. The sizes of the circles represent the sizes of the populations. The color of the circles indicates the levels of galectin-10, FOXP3, CD16, CD274 (PD-L1), IL-5R, and IL-2R expression as shown by the heat-map scale. Numbers indicate populations that are altered after treatment [1–3]. Phenotype of populations 1–3 is shown below.

**Figure 4:**
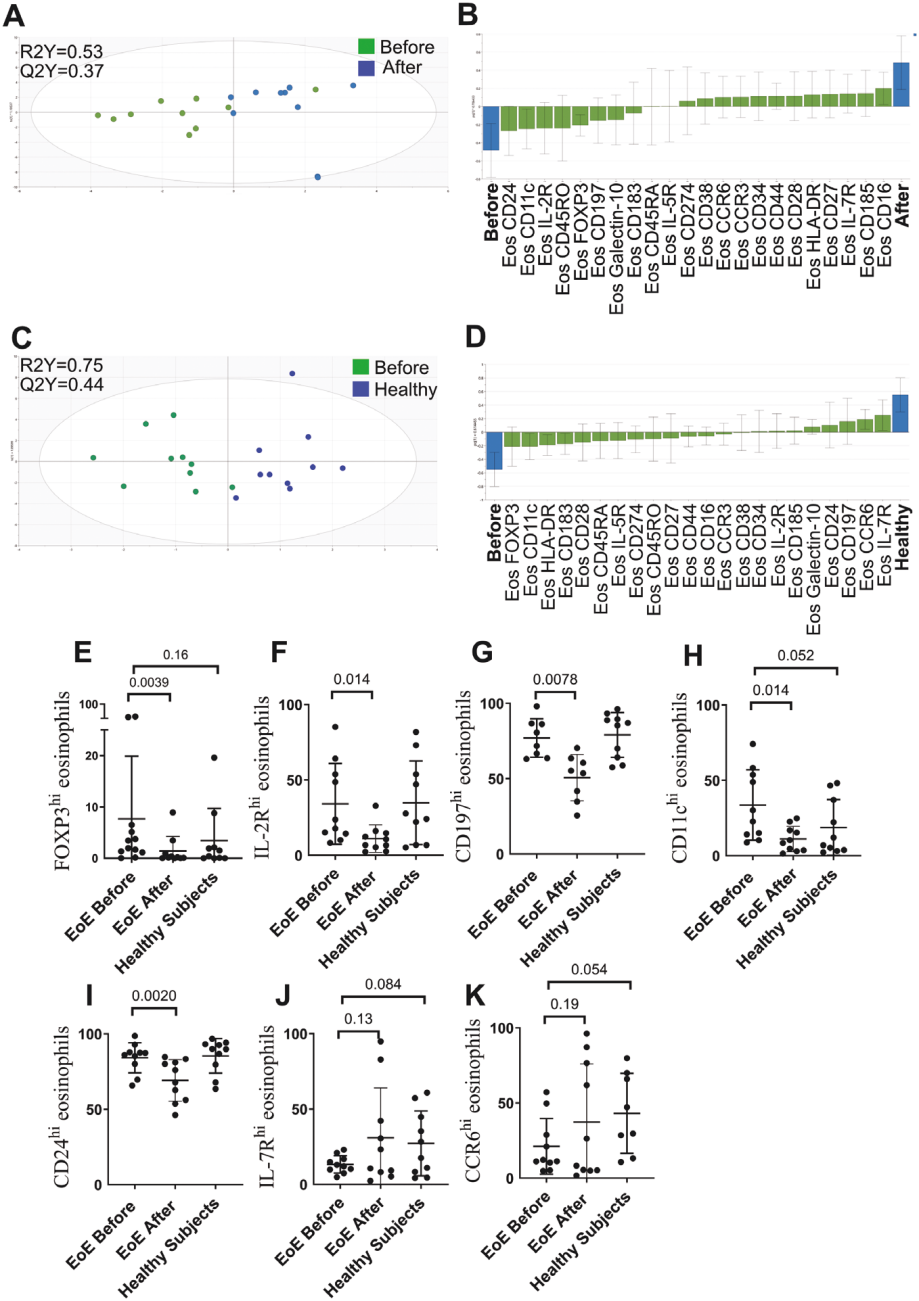
**(A)** OPLS-DA was done to see which eosinophil markers could separate EoE patients before and after treatment (*n* = 10). **(B)** Loading plots with jackknife confidence intervals for the eosinophil markers are shown as boxes with ticks. The markers closely positioned to the patient categories are positively associated to the patient category in question. The generated two-component model had an explanatory power of 53% (a goodness of fit R2Y = 0.53) and stability of 37% (Q2Y = 0.37). **(C)** OPLS-DA was done to see which eosinophil markers could separate EoE patients before treatment and healthy controls (*n* = 10). **(D)** Loading plots with jackknife confidence intervals for the eosinophil markers are shown as boxes with ticks. The markers closely positioned to the patient categories are positively associated to the patient category in question. The generated two-component model had an explanatory power of 75% (a goodness of fit R2Y = 0.75) and stability of 44% (Q2Y = 0·44). Univariate analysis of (**E**) FOXP3+ eosinophils, (**F**) IL-2R+ eosinophils, (**G**) CD197+ eosinophils, (**H**) CD11c+ eosinophils, (**I**) CD24+ eosinophils (**J**) IL-7R+ eosinophils and (**K**) CCR6+ eosinophils. Data are presented as mean ± SD.

Next, we constructed a cluster analysis of eosinophils before and after treatment with orodispersible budesonide as well as healthy controls (Fig. 3). When comparing clusters between patients before and after treatment the algorithm constructed 11 clusters in which patients before treatment had a bigger population that expressed galectin-10 (cluster 1) and FOXP3 (cluster 2). Interestingly, the intensity of galectin-10 and FOXP3 decreases after treatment. Moreover, a smaller population that expressed CD16, CD274 (PD-L1), and IL-5R (cluster 3) was seen before treatment. Phenotypes of the different eosinophil subgroups are shown below (Fig. 3, cells 1–3). Moreover, we constructed an OPLS-DA to see if we could separate the patients before and after treatment based on different eosinophil markers. A model capable of separating the two groups was generated (Fig. 3B) with a robustness of 37% (Q2Y = 0.37) and with an explanatory power of 53% (R2Y = 0.53). The most discriminatory parameters are shown in Fig. 4A. On the right are the parameters associated with patients after treatment and to the left are the parameters associated with patients before treatment. Patients before treatment had higher levels of CD24+ eosinophils, CD11c+ eosinophils, IL-2R+ eosinophils, CD45RO+ eosinophils, and FOXP3+ eosinophils and patients after treatment had higher CD16+ eosinophils, CD185+ eosinophils, IL-7R+ eosinophils, and CD27+ eosinophils (Fig. 4B). We also constructed an OPLS-DA to see if we could separate the patients before treatment and healthy controls based on different eosinophil markers. A very stable model capable of separating the two groups was generated (Fig. 4C) with a robustness of 44% (Q2Y = 0.44) and with an explanatory power of 75% (R2Y = 0.75). The most discriminatory parameters are shown in Fig. 4D. On the right are the parameters associated with healthy controls and to the left are the parameters associated with patients before treatment. Patients before treatment had higher levels of FOXP3+ eosinophils, CD11c+ eosinophils, HLA-DR+ eosinophils, CD183+ eosinophils, and CD28+ eosinophils and healthy controls had higher levels of IL-7R+ eosinophils, CCR6+ eosinophils, CD197+ eosinophils, CD24+ eosinophils, and galectin-10+ eosinophils. Finally, univariate analysis revealed that FOXP3, IL-2R, CD197 (CCR7), CD11c, and CD24 were lowered after treatment. A trend was seen that FOXP3+ eosinophils, IL-7R+ eosinophils, CD11c+ eosinophils, and CCR6+ eosinophils were higher before treatment compared with healthy controls but did not reach statistical significance (Fig. 4).

### Histological findings in the esophagus correlate with several eosinophil markers

**Figure 5:**
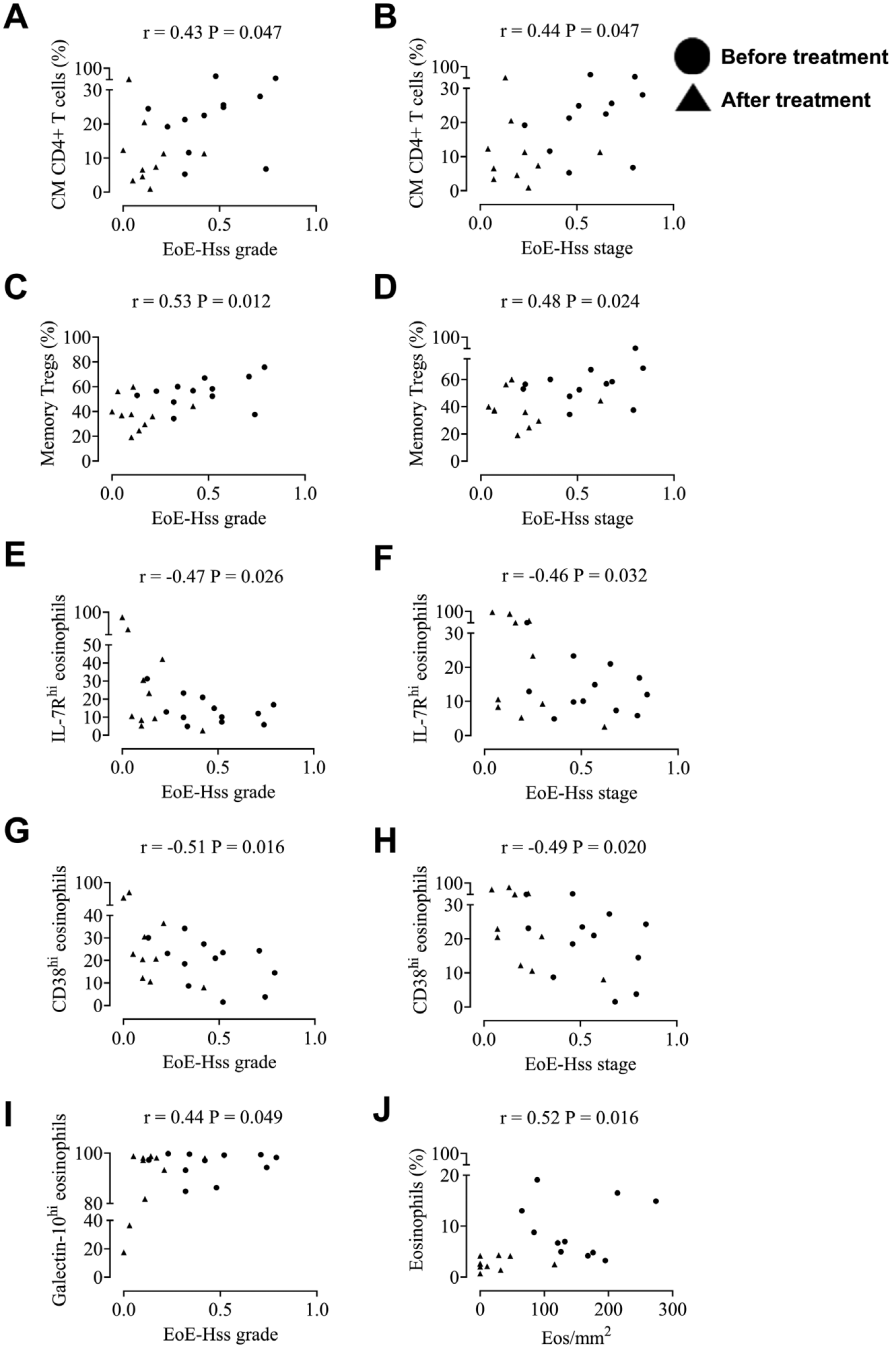
univariate analysis of the correlation between **(A)** EoE-HSS grade and central memory (CM) CD4+ T cells, **(B)** EoE-HSS stage and central memory (CM) CD4+ T cells, **(C)** EoE-HSS grade and memory Tregs, **(D)** EoE-HSS stage and memory Tregs, **(E)** EoE-HSS grade and IL-7+ eosinophils, **(F)** EoE-HSS stage and IL-7+ eosinophils, **(G)** EoE-HSS grade and CD38+ eosinophils, **(H)** EoE-HSS stage and CD38+ eosinophils, **(I)** EoE-HSS grade and galectin-10+ eosinophils and **(J)** Eos/mm^2^-and percent eosinophils. The circles are EoE patients before treatment with orodispersible budesonide and the triangles are EoE patients after treatment.

Finally, we wanted to investigate whether any immune markers correlated with EoE-HSS stage and the grade or number of eosinophils in the esophagus to find potential biomarkers in blood. Univariate analysis revealed that EoE-HSS stage and grade correlated with central memory CD4+ T cells (Fig. 5A and B), memory Tregs (Fig. 5C and D) as well as inversely with IL-7R+ eosinophils (Fig. 5E and F) and CD38+ eosinophils (Fig. 5G and H). EoE-HSS grade also correlated with galectin-10+ eosinophils (Fig. 5I) and eosinophils/mm^2^ correlated with eosinophils (Fig. 5J).

## Discussion

EoE is a chronic disease with an increasing prevalence, affecting the patient’s daily life. The patients need life-long treatment and repeated endoscopic procedures to collect esophageal tissue samples. A blood-based tool for diagnostic purposes and to monitor response to therapy is of great interest. Herein, we have investigated the major immune cell groups and, in more detail, the CD3+ T cells and eosinophils in patients with EoE before and after 12 weeks of treatment with orodispersible budesonide, 2 mg/day. In our cohort, all patients responded to the treatment although one patient did not reach remission below 15 eosinophils/HPF. This is consistent with a previous study that reported 85% of patients had achieved remission after 12 weeks [26]. We found that eosinophils decreased after treatment with orodispersible budesonide to the same level as healthy subjects, which is in line with previous reports [27]. Moreover, we saw that NK cells are decreased after treatment but there was no significant difference between EoE before treatment and healthy controls which could indicate that it is just an effect of the orodispersible budesonide and not cells involved in the pathophysiology of EoE. Even so, it is interesting that Bullock et al. demonstrated increased levels of NK cells that expressed IL-5 in pediatric EoE patients [28]. More recently a subset of NK cells producing IL-4 and IL-5 have been described in atopic dermatitis and asthma [29]. We also found that neutrophil levels were increased after treatment with orodispersible budesonide to a level of healthy subjects.

There are other reports regarding T-cell subgroups in patients with EoE with both similar and different findings compared to ours. However, noteworthy is that most studies analyze immune cells in a tissue or frozen PBMC whereas we have analyzed fresh whole blood. However, in line with our results, Eckalbar *et al*. found effector memory Th2 cells in the esophagus of EoE using single-cell RNA sequencing [30]. Morgan *et al*. found that patients with active disease had significantly higher frequencies of CRTH2+ memory CD4+ T cells (Th2 cells) in blood compared with patients in remission [31]. Herein, we can report that the effector memory CD4+ T cells were decreased after treatment to the level of healthy subjects. Our cluster analysis revealed that the effector memory Th2 cells that were lowered to the level of healthy subjects expressed IL-7R, CD44, CD5, CD28, and CD38. CD38 is a surface protein capable of inducing cell activation [32, 33], interestingly the intensity of CD38 (Th2 cells, cluster 2) was higher for the EoE patients before treatment compared with healthy subjects. Moreover, very few studies have described central memory T cells (CD197+CD45RO+) in the context of EoE. We found that both CD4+ and CD8+ central memory T cells were decreased after treatment but there was no significant difference between EoE before and healthy controls. We discovered three clusters of CD4+ central memory T cells that were Th1, Th2, and Th17 cells. Accordingly, it was the Th2 cluster that decreased after treatment. Both CD4+ and CD8+ central memory T cells expressed CD27, IL-7R, CD5, and CD28. Previous studies have shown that CD8+ T cells with a CD28+CD45RA− or CD27+CD45RA− phenotype express no or only a low level of perforin but have the ability to produce cytokines, while CD8+ T cells with a CD28−CD45RA+ or CD27−CD45RA+ phenotype express a high level of perforin but have a limited ability to produce cytokines [34, 35].

We also found that memory Tregs were decreased after treatment, although the levels before treatment were not statistically significant compared with healthy controls. Wen et al. demonstrated an enrichment of Tregs in tissue from EoE patients [3]. Abdolahi *et al*. found that children with EoE had elevated levels of Tregs in their blood compared with healthy controls [36]. In contrast, Stuck *et al*. saw decreased levels of Tregs in EoE patients with no improvement after corticosteroid treatment [37], although it was not examined if it was memory Tregs or not.

In accordance with increased levels of suppressive cells before treatment, such as Tregs, we also found that the eosinophilic phenotype was more suppressive before treatment e.g. the eosinophils expressed higher levels of the transcription factor FOXP3 and the IL-2 receptor. In addition, cluster 1 expressed high levels of galectin-10 and cluster 2 expressed high levels of FOXP3 decreased both in size and intensity after treatment. Interestingly, cluster 1 also had lower levels of the IL-5 receptor after treatment. Eosinophils in patients with EoE have elevated galectin-10 and FOXP3 levels of both mRNA and protein [19]. Eosinophils use galectin-10 in order to suppress activated T cells [38]. More recently, extracellular distribution of galectin-10 was demonstrated in the esophageal mucosa of patients with EoE which was almost absent after treatment [18]. Suppressive eosinophils that have a superior capacity to suppress activated T cells compared to conventional eosinophils express higher levels of galectin-10, CD16, CD274 (PD-L1), and CD54 (ICAM-1) than conventional eosinophils [17]. We did not see a clear difference in CD16 expression in this study, we have previously published that the CD16 expression in blood analyzed by flow cytometry was decreased after treatment with mometasone furoate aerosol [27], the reason why we did not see the same outcome in this study could be due to different methods and treatments. However, cluster 2 which was larger and with higher intensity of FOXP3 before treatment also had a decreased intensity of CD16 after treatment. Lower levels of suppressive eosinophils, after treatment with orodispersible budesonide in individuals with EoE, could indicate that activated T cells are reduced, and suppressive eosinophils are no longer needed.

Common for all three eosinophil clusters that differed the most before and after treatment, was the expression of CD197, also called CCR7. CCR7 is the chemokine receptor for CCL19 and CCL21, which attracts leukocytes to migrate toward the lymph nodes for lymphocyte interaction and antigen presentation. Moreover, CD18, an integrin beta chain protein, is lowered after treatment with mometasone furoate aerosol [39]. Accordingly, we saw that the integrin CD11c and the adhesion molecule CD24 was decreased after treatment. Indicating that eosinophils no longer need to migrate to the site of inflammation. In addition, CD11c almost reach statistical significance between EoE patients before treatment and healthy subjects. Our results point toward a less interactive, less suppressive, and less active eosinophil phenotype after treatment with orodispersible budesonide. In addition, a larger cluster of immature eosinophils expressing CD34, CD38, and IL-5R was seen after treatment (cluster 3).

An important aim of this study was to investigate if there was an immune marker that correlated with either number of eosinophils in the esophagus or the EoE-HSS. Interestingly, we found that the levels of central memory CD4+ T cells and memory Tregs correlated with EoE-HSS grade and stage. The EoE-HSS grade and stage also correlated inversely with IL-7R+ and CD38+ eosinophils. Both IL-7R and CD38 were expressed by the immature eosinophils in cluster 3. In addition, EoE-HSS grade correlated with galectin-10+ eosinophils. These results point toward the higher value of EoE-HSS the more suppressive eosinophilic phenotype and the lower value of EoE-HSS the more immature eosinophilic phenotype.

The patient-reported questionnaire WDS, was not statistically significant when comparing before and after treatment, and neither was the endoscopic EREFS score. However, the patient-reported questionnaire EEsAI reached statistical significance as did the histological evaluation, EoE-HSS, both stage and grade, and number of eosinophils/HPF. As we know patient symptoms and/or endoscopic evaluation is not always a reliable way to follow disease progression. In our cohort, many patients continue to report symptoms even though eosinophil numbers in tissue are resolved. Recent studies demonstrate the disconnect between the resolution of symptoms and the reduction in tissue eosinophil count [26, 40]. This implies how helpful and important it would be to find a blood-based diagnostic tool as a complement and hopefully even as a substitute to some of the endoscopic procedures needed to set a diagnosis and follow the disease progression. Eotaxin-3, absolute eosinophil count and eosinophil-derived neurotoxin (EDN) have been suggested as potential blood biomarkers for EoE [41]. In this study, eosinophils, effector memory CD4+ T cells, especially Th2 cells, memory Tregs, FOXP3+ eosinophils, CD11c+ eosinophils, CD38+ eosinophils, galectin-10+ eosinophils, IL-7R+ eosinophils, and CCR6+ eosinophils decrease after treatment to the level of healthy subjects or correlate with histological assessed grade of inflammation and could potentially be used as possible blood biomarkers. Some markers cannot differentiate active disease from healthy subjects even though the markers statistically change after successful treatment which could indicate that it is just an effect of the orodispersible budesonide and not cells involved in the pathophysiology of EoE. The best option based on our results seems to be eosinophils, effector memory CD4+ T cells and CD11c+ eosinophils. However, previous studies have shown that total eosinophilic count is an unreliable blood biomarker for EoE in some patients [42]. With the results from our study, the knowledge of potential biomarkers has increased.

This is the first study to monitor the immune profile of patients with EoE before and after treatment with orodispersible budesonide using CyTOF. We believe this study is an important step towards finding potential biomarkers and increasing the knowledge in the field of EoE. Still, more research is needed to be able to differentiate inflammation grade in patients with EoE based on biomarker expression. If a blood sample could be used for diagnostic purposes and/or to monitor response to therapy, the patient’s quality of life would improve substantially and these patients would not need to undergo multiple invasive endoscopic procedures. A limitation of this study is the small sample set and therefore the current findings should be viewed as exploratory and need to be confirmed in an independent larger cohort. It would also be of interest to investigate these findings in persons responding to food allergen eliminations, proton pump inhibitors, and dupilumab. In addition, we will confirm these findings in future studies in tissue samples to see if the cell phenotype correlates with the blood cell phenotype. The current findings, despite the relatively small sample, show significant differences in several different biomarkers and is of great interest.

## Supplementary data

Supplementary data is available at *Clinical and Experimental Immunology* online.

uxae065_suppl_Supplementary_Table_S1_Figure_S1

## Data Availability

The data that support the findings of this study are available from the corresponding author upon reasonable request.

## Abbreviations:

CyTOF: cytometry by time-of-flight
EEsAI: Eosinophilic Esophagitis Activity Index
EoE: eosinophilic esophagitis
EoE-HSS: EoE histology severity scoring
EREFS: eosinophilic esophagitis endoscopic reference score
NÄL: Northern Älvsborg County Hospital
WDS: Watson Dysphagia Scale
