# Supplementary material for: Investigating immune profile by CyTOF in patients with eosinophilic esophagitis after treatment with orodispersible budesonide
Source: Clin Exp Immunol. 2024 Jul 22;218(1):1–13. doi: 10.1093/cei/uxae065 (PMC11404122; doi:10.1093/cei/uxae065)
Supplement: uxae065_suppl_Supplementary_Table_S1_Figure_S1 [file uxae065_suppl_supplementary_table_s1_figure_s1.docx]

**Supplementary table S1: Antibodies used for CyTOF**

| **Antibody** | **Clone** | **Metal label** | **Source** |
| --- | --- | --- | --- |
| CD45 | HI30 | 89Y | Fluidigm |
| IgG | G18-145 | 106Cd | In house |
| RANKL | #685857 | 110Cd | In house |
| CD69 | FN50 | 111Cd | In house |
| CD31 | WM59 | 112Cd | In house |
| CD1a | HI149 | 113Cd | In house |
| CD5 | UCHT2 | 114Cd | In house |
| IgA | HP6123 | 116Cd | In house |
| CD196/CCR6 | G034E3 | 141Pr | Fluidigm |
| CD19 | HIB19 | 142Nd | Fluidigm |
| CD127/IL-7Ra | A019D5 | 143Nd | Fluidigm |
| CD38 | HIT2 | 144Nd | Fluidigm |
| IL-5R | 26815 | 145Nd | In house |
| IgD | IA6-2 | 146Nd | Fluidigm |
| CD8 | RPA-T8 | 146Nd | Fluidigm |
| CD11c | Bu15 | 147Sm | Fluidigm |
| CD274 | 29E.2A3 | 148Nd | Fluidigm |
| CD34 | 581 | 149Sm | Fluidigm |
| CD44 | IM7 | 150Nd | Fluidigm |
| CD123/IL-3R | 6H6 | 151Eu | Fluidigm |
| TCRgd | 11F2 | 152Sm | Fluidigm |
| CD185/CXCR5 | RF8B2 | 153Eu | Fluidigm |
| CD3 | UCHT1 | 154Sm | Fluidigm |
| CD45RA | HI100 | 155Gd | Fluidigm |
| CD14 | HCD14 | 156Gd | Fluidigm |
| CD27 | L128 | 158Gd | Fluidigm |
| FOXP3 | 259D/C7 | 159Tb | Fluidigm |
| CD28 | CD28.2 | 160Gd | Fluidigm |
| CD294/CRTH2 | BM16 | 161Gd | In house |
| CD66b | 80H3 | 162Dy | Fluidigm |
| CD183/CXCR3 | G025H7 | 163Dy | Fluidigm |
| Siglec-8 | 7C9 | 164Dy | Fluidigm |
| CD45RO | UCHL1 | 165Ho | Fluidigm |
| CD24 | ML5 | 166Er | Fluidigm |
| CD197/CCR7 | G043H7 | 167Er | Fluidigm |
| CD8 | SK1 | 168Er | Fluidigm |
| CD199 | L053E8 | 168Er | Fluidigm |
| CD25 | 2A3 | 169Tm | Fluidigm |
| Galectin-10 | B-F42 | 170Er | In house |
| CD20 | 2H7 | 171Yb | Fluidigm |
| IgM | MHM-88 | 172Yb | Fluidigm |
| HLA-DR | L243 | 173Yb | Fluidigm |
| CD4 | SK3 | 174Yb | Fluidigm |
| CD193/CCR3 | 5E8 | 175Lu | Fluidigm |
| CD56 | NCAM16.2 | 176Yb | Fluidigm |
| DNA1 | - | 191Ir | Fluidigm |
| DNA2 | - | 193Ir | Fluidigm |
| Cell-ID Cisplatin | - | 195Pt | Fluidigm |
| CD16 | 3G8 | 209Bi | Fluidigm |

**Supplementary figure S1.**


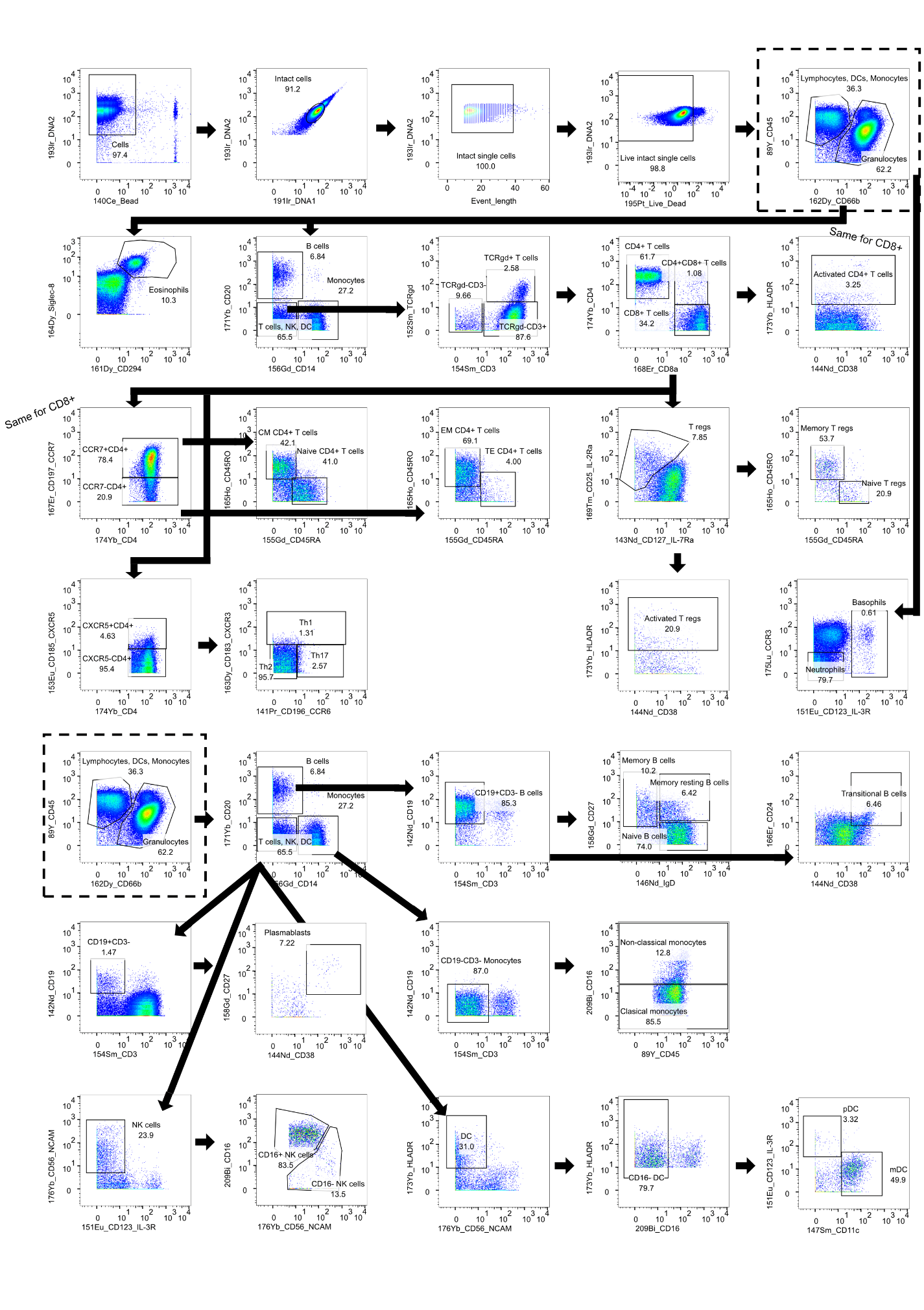


**Supplementary figure S1.** Gating strategy for samples analyzed with CyTOF. The gating is based on intact, single, live cells. Followed by gating of granulocytes from lymphocytes, dendritic cells, monocytes and NK cells. Eosinophils are gated by using CD294 and Siglec-8 from the granulocyte gate. The expression of the eosinophil markers not used in the eosinophil gating were evaluated for each sample. Gating of CD4+ T cells are depicted on the second and third row showing activated, central memory (CM), naïve, effector memory (EM), and, terminal effector (TE) CD4+ T cells, the same procedure was done for CD8+ T cells. Tregs are gated based on the expression of CD25 and CD127. Gating of Th1, Th2 and Th17 cells are shown on the fourth row and B cells on the fifth row. Rows six and seven are demonstrating the gating of plasmablast, monocytes, NK cells and dendritic cells, both myeloid (mDC) and plasmacytoid (pDC). This gating strategy has previously been published elsewhere (1).

References

1. Larsson H, Albinsson Hogberg S, Lind M, Rabe H, Lingblom C. Investigating immune profile by CyTOF in individuals with long-standing type 1 diabetes. Sci Rep. 2023;13(1):8171.
